# Self-Reported Depression, Anxiety, Stress, Health-Related Quality of Life, and Diabetes Distress During a 2-Year Pedometer-Based Physical Activity Intervention in Prediabetes and Type 2 Diabetes: Secondary Outcomes of a Randomized Controlled Trial

**DOI:** 10.1177/21501319261434134

**Published:** 2026-03-30

**Authors:** Yohannes Woldamanuel, Jenny Rossen, Therese Anderbro, Philip Von Rosen, Patrick Bergman, Maria Hagströmer, Unn-Britt Johansson

**Affiliations:** 1 Sophiahemmet University, Stockholm, Sweden; 2 Karolinska University Hospital, Huddinge, Sweden; 3 Stockholm University, Sweden; 4 Karolinska Institutet, Stockholm, Sweden; 5 Linnaeus University, Kalmar, Sweden; 6 Academic Primary Care Center, Region Stockholm, Sweden

**Keywords:** type 2 diabetes, prediabetes, physical activity, questionnaires, randomized controlled trial

## Abstract

**Background:**

Regular physical activity is essential for preventing and managing type 2 diabetes. This study evaluates self-reported outcomes including depression, anxiety, stress, diabetes distress, and health-related quality of life during a 2-year pedometer-based intervention, with or without physical activity counseling, in people with prediabetes or type 2 diabetes.

**Methods:**

This study was a 3-armed parallel randomized controlled trial conducted in primary healthcare, involving people with prediabetes or type 2 diabetes. The 3 arms were: a multi-component intervention (self-monitoring of steps with counseling support), a single-component intervention (self-monitoring of steps without counseling support), and a control group (standard care). Secondary outcomes were measured using questionnaires for self-reported depression and anxiety (the Hospital Anxiety and Depression Scale [HADS]), stress (Perceived Stress Scale [PSS]), health-related quality of life (EuroQol, [EQ-5D 3L, and EQ-VAS] ), and diabetes distress (The Problem Areas in Diabetes questionnaire, [Swe-PAID-20]), at baseline and at months 3, 6, 12, 18, and 24. Intervention effects were evaluated by a robust linear mixed model.

**Results:**

In total, 188 patients (n = 64, 59, 65 in the respective group) were included. The mean (SD) age was 64.1 (7.7) years, 21% had prediabetes, 40% were female, and HbA1c was 49.9 (11.4) mmol/mol. The retention rate was approximately 91% of participants in the first year and 81% at the end of the second year. There were no significant within-group changes in self-reported outcomes from baseline up to 24 months. Moreover, there were no significant differences in outcomes between the interventions and control group at any time in any outcome.

**Conclusion:**

This 2-year, pedometer-based physical activity intervention, despite its effectiveness for other outcomes in previous studies, is likely insufficient on its own to improve psychological well-being or reduce diabetes distress in a primary care population with prediabetes or type 2 diabetes with generally well-managed mental health at baseline. The study offers methodological insights that can guide future research. It highlights the complexity of assessing mental health outcomes within practical, low-intensity physical activity interventions.

## Introduction

Regular physical activity has been demonstrated to be an effective strategy for both the prevention and management of type 2 diabetes. Initial treatment for prediabetes and type 2 diabetes is lifestyle modification and a variety of physical activities can improve health outcomes and glycemic management. It is recommended that all individuals engage in regular physical activity, minimize sedentary time, and interrupt prolonged sitting with frequent activity breaks.^1,2^ However, research showed that individuals with prediabetes or type 2 diabetes do not achieve recommended levels of physical activity.^3,4^ Interventions that provide outcome feedback and support the integration of physical activity into daily routine, particularly those grounded in behavioral theories have been among the most effective.^5^ Furthermore, self-care interventions in primary care addressing type 2 diabetes are suggested to be effective for metabolic control.^6^

Psychological factors, such as depression and stress are possible contributors to the development of type 2 diabetes.^7^ The prevalence of depression is higher among people with type-2 diabetes compared to the general population.^8^ Moreover, a systematic review showed that people with type-2 diabetes are at an increased risk of developing anxiety symptoms compared with the general population. Depression, anxiety, or their co-occurrence are associated with an increased risk of mortality in people with diabetes,^9^ and affect diabetes self-care.^10^ High levels of perceived stress have also been linked to unemployment and the use of antidepressant medication.^11^ Studies show that older patients and those with multiple comorbidities report lower health-related quality, suggesting that the burden of managing type 2 diabetes intensifies with age and additional health complexities.^12,13^

Diabetes distress is the emotional response to living with diabetes in relation to the quality of healthcare obtained, the availability of support, coping with the emotional burden, and managing their self-care routines and its long-term complications.^14^ A systematic review and meta-analysis have found that the prevalence of diabetes distress varies across countries and affects approximately 40% of individuals with type 2 diabetes.^15^ Moreover, the co-occurrence of diabetes distress and depression is estimated to affect over 15% of this population.

Diabetes distress has been associated with lower levels of self-care, well-being, and potentially unfavorable metabolic outcomes.^16^ Therefore, recognizing and targeting diabetes distress is critical for optimal disease management in individuals with type 2 diabetes.^17^

A recent systematic review and meta-analysis showed that physical activity either alone or in combination with other interventions significantly reduces depressive symptoms in adults with type 2 diabetes.^18^ Pedometer-based interventions can substantially increase free-living physical activity,^19^ particularly when used as a motivating tool,^20^ and appear to help individuals with type 2 diabetes stay motivated and maintain their activity levels over time.^21^

The Sophia Step Study, a 2-year, 3-armed randomized controlled trial (RCT), evaluated the effects of a pedometer-based intervention with and without counseling in individuals with prediabetes or type 2 diabetes.^22,23^ The process evaluation study demonstrated that a pedometer-based intervention both with and without counseling are feasible methods to increase and maintain daily steps.^23,24^ Furthermore, participants characterized by lower baseline step counts, lower age, male gender, or higher self-efficacy tended to achieve an increase of more than 500 steps per day in response to the intervention.^25^ Several secondary outcome measures have not been explored thus far in this RCT. This study evaluates self-reported outcomes including depression, anxiety, stress, diabetes distress, and health-related quality of life during a 2-year pedometer-based intervention, with or without physical activity counseling, in people with prediabetes or type 2 diabetes.

## Methods

### Study Design

The study was a 3-armed randomized controlled trial conducted over 2 years in Sweden, targeting individuals with prediabetes or type 2 diabetes in a primary healthcare setting. Detailed information can be found in the published study protocol.^22^ Effects on the primary outcome, HbA1c, and other secondary outcomes such as cardiometabolic risk factors and objectively measured physical activity have been reported elsewhere.^24^ The Consolidated Standards of Reporting Trial Guidelines (CONSORT),^26^ was used for reporting the study.

### Participant Recruitment

Participants were recruited by their diabetes specialist nurses from the primary healthcare centers they visited. Approximately 385 participants were invited for enrollment during 8 rounds between April 2013 and January 2018 from 2 urban and 1 rural area primary healthcare centers in Sweden. The invitation occurred during a regular face-to-face visit to primary healthcare centers or by phone calls and participants were randomized into 3 arms. Project staff in the project prepared sealed envelope stratified by gender to keep the randomization allocation ratio at 1:1:1. The diabetes specialist nurse distributed the envelopes in the order of the participants’ first visit.^22^

The inclusion criteria were ≥1 year duration of type 2 diabetes, or prediabetes with HbA1c >39 to <47 mmol/mol and/or fasting blood glucose >5.6 mmol/L, an age of 40 to 80 years, and capacity to read, write, and communicate in Swedish language.

The exclusion criteria were myocardial infarction within the past 6 months, serum creatine levels exceeding 140 µmol/L, presence of diabetic foot ulcers or severe peripheral neuropathy, initiation of insulin treatment within the past 6 months, any condition prohibiting physical activity, history of severe hypoglycemia within the past 12 months, participation in very high-intensity activities as per the Stanford Brief Activity Survey,^27^ and lacking access to the internet.

### Intervention Description

A 2-year intervention was implemented within primary health care settings to encourage regular physical activity among people with prediabetes or type 2 diabetes. In the study protocol details of the intervention and theoretical framework are described.^22^ Step counters (Yamax Digiwalker SW 200: Yamax Corporation, Tokyo, Japan) together with instructions to self-report their daily steps on a website were given by the diabetes specialist nurse to participants of the 2 intervention groups: a multi-component intervention (self-monitoring of steps with counseling support) and a single-component intervention (self-monitoring of steps without counseling support). Participants in the multi-component intervention group received step counters and instructions to self-report their daily steps on to a website where they could track their progress, 12 group counseling sessions (10 completed within the first year of the intervention) led by project staff (the urban centers) and a diabetes specialist nurse (rural center). Furthermore, the participants received 9 individual face-to-face counseling sessions (7 completed within the first year) by their diabetes specialist nurse. The single-component intervention group received only step counters and instructions to self-report their daily steps to track their progress by their diabetes specialist nurse. The program for the group sessions was guided by the health belief model,^28^ social cognitive theory,^29^ and the transtheoretical model of change,^30^ and considered several techniques for behavior change.^22^ Individual consultations were based on a motivational interviewing technique.^31^ The diabetes specialist nurses (n = 3) were conventionally trained in motivational interviewing, in the applied behavior change theories and in the method to prescribe physical activity. The third group served as a control group and received standard care including meeting with a diabetes specialist nurse and a physician once a year, or more often if needed.

### Data Collection and Outcomes

Demographics were collected at baseline by a questionnaire to obtain information about age, gender, university education, and marital status and participants’ data on health conditions was obtained from the medical records.

The participants completed a battery of questionnaires at baseline, and at 3, 6, 12, and 24 months to measure self-reported depression, anxiety, stress, health related quality of life and diabetes distress for the analysis of secondary outcome data for the Sophia Step Study. Depression and anxiety were measured with the Hospital Anxiety and Depression Scale (HADS).^32^ The scale is designed to measure depression and anxiety in 2 combined subscales, with 7 questions related to depression (HAD-D) and 7 associated with anxiety (HAD-A). Each subscale has 7 items with a 4-point Likert scale (0-3). The total score for each subscale adds up to 21. For example, the “no anxiety/depression” score is between 0 and 7, the score for “moderate anxiety/depression” lies between 8 and 10, and finally, a score >10 is considered “severe anxiety/depression.”^33^ Stress was measured with the Perceived Stress Scale (PSS), which measures the degree of the perceived stress in the person’s life. It has 14 items to which the respondents rate their perceived stress status on a 5-point scale (0 = never and 4 = very often) for the past 1-month. The Swedish version of the PSS is used in the current study; the instrument is valid and reliable for measuring mental stress.^34^ Diabetes distress,^35^ was measured using The Problem Areas in Diabetes questionnaire (Swe-PAID-20).^36^ The Swe-PAID instrument has 20 items that produce a total score ranging from 0 to 100. A higher score implies more diabetes-related emotional distress. The items in Swe-PAID have a 5-point Likert scale in which 0 = not a problem to 4 = serious problem. The total score can be obtained by multiplying the total rate by 1.25. It was proven to be valid and reliable for the Swedish diabetes population.^36^ The cut-off score is suggested to be ≥ 40.^35^ Health-related quality of life was measured using the EuroQol (EQ-5D-3L),^37^ and combines 2 parts called the EQ-5D of the descriptive system and the EQ visual analog scale (EQ-VAS). The EQ-5D contains 5 dimensions: mobility, usual self-care activities, pain/comfort, and anxiety/depression with a 3-level rating, that is, no problem, some problem, and extreme problem. The EQ-VAS has 2 endpoints that extend on a vertical scale from ‘Best imaginable health state’ (100) to ‘Worst imaginable health state’ (0).^38^

### Statistical Analysis

An intention-to-treat approach was applied, and the effects of the intervention were analyzed as follows. First, the distribution was examined visually for each outcome, where the values were plotted over time. All the outcomes had outliers with a certain degree of floor/ceiling effects in which the values were gathered at the minimum or maximum of the scale. The impact of the treatment (single- or multicomponent groups or control group) and time (baseline, 3, 6, 12, and 24 months) were analyzed using robust linear mixed models for each of the outcomes (depression, anxiety, stress, health-related quality of life, and diabetes distress).^39^ Models were fitted with random effects for subject ID and fixed effects for baseline value, group, time, and group-by-time interaction. The coefficient and *P*-value testing indicated the effect of the intervention from baseline to different outcome assessment time points. The estimated treatment differences at each time point were extracted from the models, that is, using a 95% confidence interval and the *P*-value (.05). The mixed models were used to fit all time points in the same model and to account for missing data. We used the software R version 4.1.1 to analyze the data.^40^ The statistical analysis was conducted by an independent statistician who was blinded to group allocation.

## Results

**Figure 1.**
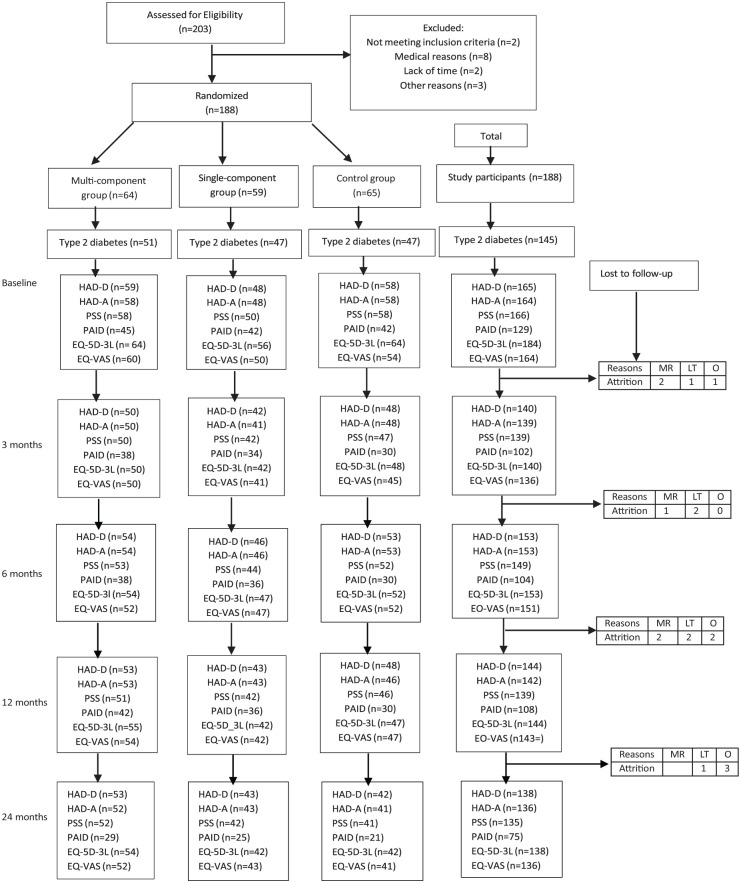
Flow diagram of the study population and each measurement point. Abbreviations: MR, medical reason; LT, lack of time; O, others.

Figure 1. presents a flow diagram illustrating participant drop-out at each measurement point throughout the trial. Three hundred eighty-five patients from the 3 primary healthcare centers were invited to participate in the Sophia Step Study, and 203 agreed to participate. Out of the 203 individuals who agreed to participate, 15 withdrew or were excluded before randomization due to medical reasons, lack of time, not meeting the criteria, and other reasons. Therefore, 188 participants were allocated to 3 different intervention levels, 64 were assigned to the multiple-component group, 59 were assigned to the single-component group, and 64 were assigned to the control group. The retention rate was approximately 91% of participants in the first year and 81% at the end of the second year. However, as shown in Figure 1, the response rate of self-reported depression (HAD-D), anxiety (HAD-A), stress (PSS), diabetes distress (Swe-PAID), and health-related quality of life (EQ-5D 3L and EQ-VAS) varied between collection points and among the groups. The figure presents the valid participants’ response rate of the questionnaires (HAD-D, HAD-A, PSS, Swe-PAID, EQ-5D 3L, and EQ-VAS) and dropouts at each point in time throughout the intervention periods during data collection.

Baseline characteristics are presented in Table 1. At baseline, the participants’ mean age was 64.1 (SD = 7.7) years, and 40% were women. A total of 20% of participants were diagnosed with prediabetes, 46% had higher education, and 66% lived with a partner. The mean BMI was 30.0 (SD = 4.4) kg/m^2^, and the mean HbA1c level was 49.9 (SD = 11.4) mmol/mol. At baseline, self-reported depression (HAD-D), anxiety (HAD-A), stress (PSS), diabetes distress (Swe-PAID), and health-related quality of life (EQ-5D 3L and EQ-VAS) were similar across all groups, with most participants’ scores falling within the normal range for the instruments used. Only a few outliers were noted in each group (Table 1).

**Table 1.** Baseline Characteristics and Self-reported Outcomes.

Characteristics	Overall group (n = 188)	Multi-component intervention (n = 64)	Single-component intervention (n = 59)	Control group (n = 65)
Demographics				
Age (years), mean (SD)	64.1 (7.7)	64.2 (6.9)	65.1 (7,3)	63.1 (68.7)
Female, n (%)	76 (40.4)	28 (43.8)	24 (40.7)	24 (36.9)
Prediabetes, n (%)	40 (21.2)	13 (20.3)	10 (16.9)	17 (26.2)
Diabetes duration in years, mean (SD)	8.2 (5,8)	9.4 (7.1)	7.9 (5.2)	7.3 (4.8)
Prediabetes duration in years, mean (SD)	1.9 (1.8)	1.6 (1.4)	2.1 (2.2)	1.9 (1.6)
University education, n (%)	87 (46.3)	28 (43.7)	25 (42.4)	34 (52.3)
Living with a partner, n (%)	124 (66.0)	46 (71.9)	37 (62.7)	41 (63.1)
Cardiovascular risk factors				
HbA1c (mmol/mol), mean (SD)	49.9 (11.4)	49.4 (11.2)	50.5 (11.2)	49.9 (12.0)
BMI (kg/m^2^), mean (SD)	30.0 (4.4)	30.3 (4.19	29.4 (4.4)	30.2 (4.8)
Self-reported outcomes				
HAD-D, median (range)	3.0 (0-14)	3.0 (0-14)	3.0 (0-13)	3.0 (0-12)
HAD-A, median (range)	3.0 (0-17)	3.0 (0-12)	3.0 (0-11)	3.0 (0-18)
PSS, median (range)	20.0 (5-50)	20.5 (5-34)	19.0 (6-45)	21.0 (5-50)
Swe-PAID-20, median (range)	15.0 (0-50)	13.0 (0-41)	15.0 (0-42)	16.0 (0-50)
EQ-5D 3L, median (range)	79.6 (30-100)	79.6 (46-100)	79.6 (66-100)	79.6 (30-100)
EQ-VAS, median (range) %	76.0 (0-100)	75.0 (0-97)	80.0 (32-100)	76.5 (33-97)

As shown in Tables 2 to 4 there were no statistically significant differences in mean scores for depression and anxiety, stress, diabetes-related distress, or health-related quality of life between the intervention and control groups at any time point. Furthermore, no notable differences were detected among the intervention groups across the 3-, 6-, 12-, and 24 months follow-up periods.

**Table 2.** Intervention Effects on Self-reported Depression (HAD-D), Anxiety (HAD-A), Stress (PSS), Diabetes Distress (Swe-PAID), and Health-related Quality of Life (EQ-5D 3L and EQ-VAS) at the Follow-ups to 24 Months Between the Multi-component Intervention and Control Group.

Multi-component intervention vs control group	Diff. (95% CI) 3 months	Diff. (95% CI) 6 months	Diff. (95% CI) 12 months	Diff. (95% CI) 24 months
HAD-D	0.01 (−0.71, 0.72)	−0.02 (−0.71, 0.67)	0.29 (−0.42, 1.00)	0.62 (−0.10, 1.34)
HAD-A	−0.02 (−0.77, 0.74)	0.43 (−0.30, 1.15)	0.52 (−0.24, 1.28)	0.71 (−0.06, 1.49)
PSS	−0.26 (−2.39, 1.87)	−0.02 (−2.08, 2.04)	0.48 (−1.68, 2.63)	1.06 (−1.10, 3.23)
Swe-PAID-20	0.53 (−3.63, 4.69)	−1.80 (−5.95, 2.36)	−1.03 (−5.21, 3.14)	1.27 (−3.29, 5.84)
EQ-5D 3L	−0.49 (−3.69, 2.71)	−0.46 (−3.60, 2.68)	−3.06 (−6.24, 0.11)	−0.80 (−4.04, 2.45)
EQ-VAS	−0.12 (−5.38, 5.14)	5.67 (0.63, 10.71)	3.03 (−2.16, 8.22)	1.72 (−3.57, 7.01)

Abbreviations: VAS, visual analog scale; Diff., difference.

**Table 3.** Intervention Effects on Self-reported Depression (HAD-D), Anxiety (HAD-A), Stress (PSS), Diabetes Distress (Swe-PAID), and Health-related Quality of Life (EQ-5D 3L and EQ-VAS) at the Follow-ups to 24 Months Between the Single-component Intervention and Control Group.

Single-component intervention vs control group	Diff. (95% CI) 3 months	Diff. (95% CI) 6 months	Diff. (95% CI) 12 months	Diff. (95% CI) 24 months
HAD−D	−0.20 (−0.96, 0.56)	−0.33 (−1.07, 0.41)	−0.15 (−0.91, 0.60)	−0.22 (−0.99, 0.55)
HAD−A	0.34 (−0.47, 1.14)	0.21 (−0.55, 0.97)	0.20 (−0.60, 1.00)	0.06 (−0.76, 0.89)
PSS	−0.43 (−2.67, 1.80)	−0.94 (−3.11, 1.24)	0.52 (−1.71, 2.76)	−0.07 (−2.36, 2.23)
Swe−PAID−20	−0.96 (−5.23, 3.30)	2.87 (−1.37, 7.11)	−0.29 (−4.57, 4.00)	−2.10 (−6.79, 2.59)
EQ−5D 3L	2.83 (−0.52, 6.17)	2.38 (−0.88, 5.64)	3.47 (0.11, 6.83)	3.40 (−0.01, 6.81)
EQ−VAS	1.31 (−4.23, 6.85)	−0.31 (−5.56, 4.95)	0.88 (−4.59, 6.34)	1.14 (−4.46, 6.74)

Abbreviations: VAS, visual analog scale; Diff., difference.

**Table 4.** Intervention Effects on Self-reported Depression (HAD-D), Anxiety (HAD-A), Stress (PSS), Diabetes Distress (Swe-PAID), and Health-related Quality of Life (EQ-5D 3L and EQ-VAS) at the Follow-ups to 24 months Between the Multi-component Intervention and Single-component Intervention.

Multi-component intervention vs single-component intervention	Diff. (95% CI) 3 months	Diff. (95% CI) 6 months	Diff. (95% CI) 12 months	Diff. (95% CI) 24 months
HAD-D	−0.19 (−0.94, 0.55)	−0.35 (−1.08, 0.38)	0.14 (−0.59, 0.87)	0.40 (−0.35, 1.14)
HAD-A	0.32 (−0.47, 1.11)	0.63 (−0.13, 1.40)	0.72 (−0.05, 1.49)	0.78 (−0.01, 1.57)
PSS	−0.69 (−2.91, 1.53)	−0.96 (−3.12, 1.21)	1.00 (−1.18, 3.19)	1.00 (−1.22, 3.21)
Swe-PAID-20	−0.43 (−4.53, 3.66)	1.80 (−2.94, 5.09)	−1.32 (−5.32, 2.68)	−0.83 (−5.18, 3.52)
EQ-5D 3L	2.34 (−0.98, 5.65)	1.92 (−1.31, 5.15)	0.40 (−2.87, 3.68)	2.60 (−0.68, 5.89)
EQ-VAS	1.19 (−4.11, 6.50)	5.36 (0.24, 10.49)	3.90 (−1.26, 9.06)	2.86 (−2.41, 8.14)

Abbreviations: VAS, visual analog scale; Diff., difference.

## Discussion

This study evaluated self-reported depression, anxiety, stress, diabetes distress, and health-related quality of life over a 2-year pedometer-based intervention including self-monitoring of daily steps with or without physical activity counseling in people with prediabetes or type 2 diabetes. The results indicated no statistically significant improvements in these self-reported secondary outcomes.

Notably participants’ baseline scores were within the normative range and suggesting absence of clinically relevant symptoms at the outset which may have limited the potential for observable improvements. However, a meta-analysis revealed that physical activity interventions could reduce depressive symptoms even among those without clinical diagnosis of depression.^18^ A similar study involving individuals with or at risk of developing type 2 diabetes implemented a 10-week intervention combining cognitive mentoring sessions with an outdoor exercise circuit challenge using a smartphone application. This intervention resulted in a reduction in depressive symptoms.^41^ The inconsistency in findings between the studies may be attributable to the use of different questionnaires for assessing depressive symptoms, different intervention components, or a higher number of study participants experiencing symptoms of depression at baseline.

A previous narrative review noted that most studies have examined mental health outcomes as secondary or exploratory variables rather than as primary outcomes of interventions.^42^ Furthermore, the diversity in patient reported outcome measures used across studies is a challenge to the validity and comparability of results from different studies. The lack of standardization and harmonization in outcome measures emphasizes the need for greater consistency in the selection and application of patient reported outcome measures in diabetes research.^43^

To comprehensively evaluate the effectiveness of an intervention, it is essential to incorporate the patient’s perspective alongside clinical and physiological outcomes. This approach not only assesses the efficacy of the intervention but also ensures that there is no deterioration in perceived health status. Patient-reported outcome measures are particularly valuable in this context as they offer guidance to people with diabetes about the expected course of disease and treatment, for shared decision making, evaluate outcomes and strengthen healthcare.^43^ A previously qualitative interview study exploring participants’ experiences of the Sophia Step Study found that all 3 intervention groups appreciated the study assessment. The participants reported that receiving feedback on health outcomes, was encouraging, gave a sense of taken care of and were adequately inspirational.^44^ These findings underscore the importance of integrating patient-reported outcomes into intervention studies, not only as evaluative tools but also to enhance participant engagement and motivation.

In this study we used 3 generic instruments (HADS,^32^ PSS,^34^ and EQ-5D 3L and EQ-VAS^37^) and 1 disease specific instrument (Swe-PAID-20^36^).

To ensure a comprehensive assessment of treatment outcomes, it is important to incorporate both generic and disease-specific self-reported outcome measures. While these instruments capture the patients’ perspectives, they may not be sensitive enough to detect clinically meaningful changes specific to the condition in RCT investigation.^43^ Therefore, careful consideration must be given when interpreting results from patient-reported outcome measures in RCTs. One possible explanation for the absence of significant differences between groups is the presence of ceiling or floor effects. These effects occur when many participants score near the upper or lower end of the scale thereby limiting the ability to detect further improvements or deterioration. As described by Andrade,^45^ these effects can mask true differences between treatment groups in an RCT, especially if both the intervention and control groups reach near-maximum or minimum values on the scale.

Furthermore, both the multi-component and single-component intervention primarily designed to promote physical activity using models and techniques for behavior change. The intervention did not specifically focus on psychological aspects of living with type 2 diabetes.^22^ Previous systematic reviews have reported that physical activity interventions is more effective in improving mental health when combined with other interventions, such as psychological therapies.^46,47^

This study has several noteworthy strengths. First, the use of a randomized controlled study design, combined with long intervention duration and repeated measures of self-reported depression, anxiety, stress, health-related quality of life, and diabetes distress. This helps to reduce potential bias and minimize confounding factors that might have affected the results. Additionally, the use of linear mixed models to analyze longitudinal outcomes of repeated measures provides robust estimation of the outcomes over time without the need to impute missing data. The statistical approach strengthens the reliability of the findings by appropriately handling repeated measures and account for individual variability. Another strength was the utilization of reliable and validated generic and disease specific questionnaires. This approach allows for a better comprehension of the health outcomes in individuals with type-2 diabetes. To reduce seasonal variation, the recruitment was conducted during autumn, winter, and spring. Both genders were included and with a majority of men.

The current study has some limitations that need to be addressed. First and foremost, this analysis represents a secondary outcome evaluation of the Sophia Step Study, which included both participants with prediabetes or type 2 diabetes with minimal diabetes complications and received regular health check-ups at their respective primary healthcare centers. As a result, most participants overall health scores comparable to the general population, which may have limited the potential to detect significant improvements in mental health status. Moreover, the study was powered based on the primary outcome HbA1c rather than the secondary outcomes. As such, the analyses of the secondary outcomes may have been underpowered reducing the likelihood to detecting significant effects. Additionally, no data was gathered regarding the participants’ use of psychiatric medication before or during the study period which may have introduced unmeasured confounding. Third, caution should be exercised when attempting to generalize the current study’s findings to the wider population of individuals with prediabetes or type 2 diabetes. The sample size drawn from only 2 geographical locations in Sweden limiting the external validity. Participants in this study were not blinded because the intervention included individual and group counseling sessions, making blinding impractical. Additionally, all 3 groups were recruited from the same primary healthcare centers, increasing the risk of intervention contamination. Finally, although the study was not originally powered to assess self-reported health outcomes, the analysis provides exploratory insights into the relationship between self-reported depression, anxiety, stress, health-related quality of life, and diabetes distress and prediabetes and type 2 diabetes.

## Conclusion

This 2-year, pedometer-based physical activity intervention, despite its effectiveness for other outcomes in previous studies, is likely insufficient on its own to improve psychological well-being or reduce diabetes distress in a primary care population with prediabetes or type 2 diabetes with generally well-managed mental health at baseline. The study offers methodological insights that can guide future research. It highlights the complexity of assessing mental health outcomes within practical, low-intensity physical activity interventions.
